# Ultramicroelectrode Array Based Sensors: A Promising Analytical Tool for Environmental Monitoring

**DOI:** 10.3390/s100100475

**Published:** 2010-01-07

**Authors:** Jahir Orozco, César Fernández-Sánchez, Cecilia Jiménez-Jorquera

**Affiliations:** Instituto de Microelectrónica de Barcelona, IMB-CNM (CSIC), Campus UAB, 08193 Bellaterra, Barcelona, Spain; E-Mails: cesar.fernandez@imb-cnm.csic.es

**Keywords:** ultramicroelectrode array, microsensor, analytical tool, environmental monitoring

## Abstract

The particular analytical performance of ultramicroelectrode arrays (UMEAs) has attracted a high interest by the research community and has led to the development of a variety of electroanalytical applications. UMEA-based approaches have demonstrated to be powerful, simple, rapid and cost-effective analytical tools for environmental analysis compared to available conventional electrodes and standardised analytical techniques. An overview of the fabrication processes of UMEAs, their characterization and applications carried out by the Spanish scientific community is presented. A brief explanation of theoretical aspects that highlight their electrochemical behavior is also given. Finally, the applications of this transducer platform in the environmental field are discussed.

## 1. Introduction

In the 1970s the discovery, although non elucidation, of the unusual properties of ultramicroelectrodes (UMEs) opened new possibilities of analyzing electrode processes. The changes in mass transport conditions bring about extremely high current densities at UMEs, whereas the current themselves become very small [1]. One decade later the first comprehensive survey of the special properties and perspectives of the so-called micro-voltammetric electrodes was provided [2]. Since then, the concept of UME has been extended in the literature and can be defined taking into account that only one of the electrode characteristic dimensions, given by the geometry, must be in the order of some micrometers. Such critical dimension makes the electrochemical response differ from that of a conventional electrode and opens up new possibilities for studying electrode reactions, which includes dynamic measurements in solutions with low electrolyte concentration, in non-polar solvents or low conducting media, and even in the solid phase or gas state [3]. Furthermore the use of UMEs expands the time scale for carrying out measurements by several orders of magnitude, which is particularly useful to study rapid homogeneous or heterogeneous reactions. It is the possibility of achieving responses in the steady-state what makes UMEs highly attractive for specific applications. This feature is related with the equilibrium established between the diffusion of electroactive species that arrive to the electrode surface due to the concentration gradient and the electroactive species that exchange electrons at this surface. Such an equilibrium is not possible at bigger electrodes, where the amount of species exchanging electrons at their surface is higher than those diffusing from the bulk of the solution, thus attaining transient currents. As a consequence UMEs can be applied in highly resistive media and in very fast scan-rate voltammetric experiments, oxygen does not interfere in the electrochemical experiments and the signal is less dependent on convection. Likewise, their small size is a key factor in specific applications such as single brain cells or capillary chromatography detectors [4–6].

The very small currents measured with individual UMEs have led to the development of ensembles of ultramicroelectrodes working in parallel, with which current amplification is attained. When these ensembles are formed by *m* identical microelectrodes evenly distributed in an array, the resulting configuration is termed ultramicroelectrode array (UMEA). Ideally, these arrays should yield a current amplification by a factor of *m* relative to a single microelectrode [7]. In this context, UMEAs are being used for numerous applications including biochemical and environmental analysis [8–11], particularly as analytical screening tools for heavy metal detection [8,12–17].

Regarding chemical tools of analysis, over the last half century the design and development of analytical protocols and instrumentation have experienced important changes due to the growing demand for more and high-quality (bio)chemical information [18]. It is the environmental field one of the main research areas where new and high-performance analytical approaches have been explored for a huge variety of different applications. Monitoring different parameters of environmental interest usually requires carrying out rapid on-site measurements with high reproducibility, good sensitivity and very low limits of detection. Additionally, the development of user-friendly approaches is very demanding. However, most analytical devices currently employed for routine analysis use bulky and expensive instrumentation, have to be performed by highly specialized personnel in centralized laboratories and are time-consuming.

In this context, many research groups across the world have focused their research efforts in the development of miniaturized devices that meet all the above-mentioned demands, trying to highlight the potential that such approaches exhibit for specific analytical environmental applications. The common trend towards the miniaturization of analytical devices have led to the development of sensors of more and more smaller dimensions, which are also required to be robust, have low output impedance, rapid response and to be mass-produced at a reasonable cost. Their size is crucial if they are to be implemented in small probes designed for in-field measurements [19,20] or integrated in flow systems were sample consumption is drastically reduced [21].

In this work, ultramicroelectrode arrays (UMEAs) based devices developed by different research groups in Spain for the detection of different parameters of environmental interest have been reviewed. Work presented is referred to UMEAs as large numbers of UMEs fabricated on the same substrate and connected in parallel, whose electrochemical response corresponds to the sum of the electrochemical response of the individual microelectrodes under suitable separation/width ratio, depth and time window conditions. Theoretical considerations that explain their electrochemical behavior as well as their fabrication and characterization processes are summarized. In addition, several approaches for the sensitive and selective detection of chemical species, whose performance rely on the use of nanoparticles and/or biomolecules are described.

## 2. Theoretical Considerations

Ultramicroelectrode (UME) is a term used to describe microelectrodes where at least one of their dimensions is smaller than the thickness of the target analyte diffusion layer. When this happens, the electrode voltammetric performance is dramatically enhanced because of: a) Improved mass transport towards the transducer (radial diffusion dominates); B) reduced double-layer capacitance and thus greatly enhanced faradaic to capacitive current ratios; and C) very small *iR* drop. Ultramicroelectrodes of planar configuration in a range of configurations, from discs to bands, have been developed using microfabrication technologies. However, due to the very small currents measured with these devices, ensembles of ultramicroelectrodes have been designed as a means of increasing the magnitude of the current while retaining those advantages of a single UME mentioned above. The term ultramicroelectrode array (UMEA) is referred to a device formed by *m* identical microelectrodes. Ideally, these arrays should yield a current amplification by a factor of *m* relative to a single microelectrode [7]. In practice, several requirements should be met in order to attain this signal amplification. One of them is related to the packaging density of microelectrodes. It has been assessed that loosely packed arrays where the inter-electrode distance *d* ≫ 2*r* (*r* being the radius of a single microelectrode) yield the expected current signal (*m* times amplified) whereas closely packed arrays, where *d* ≈ 2*r*, behave as a macroelectrode having a current that is proportional to the total geometric area of the microelectrodes in the array. For example, Belmont *et al.* suggested that separation distances should be at least 10 times the diameter of an individual microelectrode [12].

**Figure 1.**
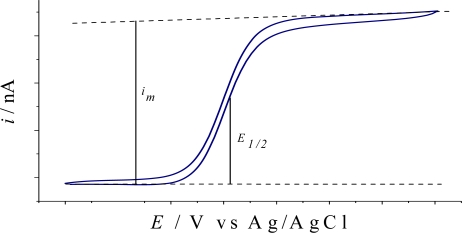
Typical sigmoidal signal obtained with an ultramicroelectrode array.

Figure 1 show a steady-state voltammogram recorded with an UMEA device. A symmetrical sigmoidal response is observed, from which half-wave potential of the electroactive species *(E_1/2_)* can be estimated [3,22]. The current recorded with a UMEA is given by the sum of steady-state currents of individual microelectrodes and can be calculated using the following equation:

(1) $$i_{m}=i\;m=4\;m\;n\;F\;D\;C\;r$$

where *i_m_*, is the steady state current of the array; *i*, is the steady state current of an individual microelectrode; *m*, is the number of microelectrode discs; *n*, is the number of electrons transferred in the redox reaction; *F*, is the Faraday constant; *D*, the analyte diffusion constant, *C* is the analyte concentration and *r*, is the radius of each microelectrode in the array.

**Figure 2.**
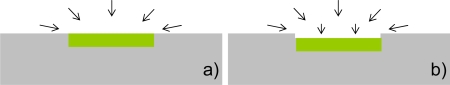
Scheme of the diffusion regimes of an analyte toward the surface of, a) inlaid, b) recessed utramicroelectrodes.

Depending on the UMEA fabrication process, either inlaid or recessed electrodes can be obtained and slight variations in the diffusion regime observed (Figure 2). Equation 1 should be used assuming that inlaid microelectrodes are present and radial diffusion of electroactive species applies, as depicted in Figure 2a. In the case of recessed UMEAs, a variation of the diffusion regime has to be considered (Figure 2b). The current recorded in this case decreases by a factor of *π* / [(4*L*/*r*) + *π*] compared with the value obtained from equation 1. Some recently published papers analyze the electrochemical performance of recessed UMEAs [23,24]. Also, other geometries and configurations such as multifunctional microelectrodes arrays [25] and nanoarrays [26] have been reported.

## 3. Ultramicroelectrode Array Fabrication Processes

Random and ordered arrays of ultramicroelectrodes have been designed and fabricated using different technologies. Screen-printing processes have given rise to the cost-effective and mass-production fabrication of ordered disk ultramicroelectrode arrays, this technique being easily implemented in a research laboratory. Random assemblies of micro-disks connected in parallel are another simple and cheap approach used to fabricate arrays. Other UMEA approaches are based on the dispersion of nanoparticles over electrode surfaces or the deposition of electrode materials within microporous structures [27].

The fabrication of UMEA-based approaches in Spain has just been carried out by the groups in the Instituto de Microelectrónica de Barcelona (IMB-CNM). Photolithographic-based processes are commonly used worldwide for the fabrication of UMEAs of planar configuration in spite of requiring expensive instrumentation and trained staff. However, this technique is very reliable as it gives rise to UMEA with perfectly controlled geometric features, which in turn can be scaled up and mass-produced for miniaturization and batch fabrication purposes, respectively [28–30]. In this context, different UMEAs designs have been fabricated at the Clean Room facilities of the IMB-CNM.

**Figure 3.**
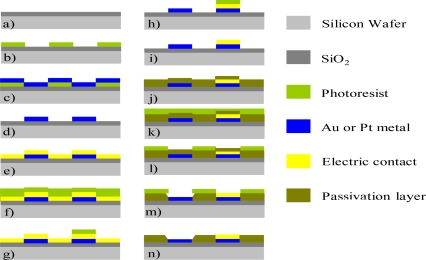
Fabrication process of a microelectrode using a standard photolithographic technique.

Au and Pt ultramicroelectrode arrays with different geometries have been fabricated according to standard photolithographic techniques using Si/SiO_2_/metal structures. A scheme of the fabrication process is depicted in Figure 3. A 4-inch diameter silicon wafer is used as substrate. A 1,000 nm-thick thermal oxide layer is firstly grown on the silicon wafer in order to avoid short-circuits with the subsequent metal layer (Figure 3a). This metal layer is defined by photolithography as follows. The wafer is spin-coated with a photoresist and exposed to UV light through a mask containing the pattern for the electrodes and interconnects (Figure 3b). A 20 nm-thin layer of Ti or Cr, as intermediary adhesion layers, followed by 100 nm-layers of Pt or Au, respectively, is deposited by e-beam evaporation (Figure 3c) as previously reported for a standard photolithographic fabrication process [31]. Next, a lift-off process is carried out to remove the photoresist and obtain the desired metal patterns (Figure 3d). In the next steps, the formation of the gold contacts is carried out. A thin gold layer is deposited by sputtering (Figure 3e) and contacts patterned by a second photolithographic process (Figures 3f and 3g). A wet etching process is then carried out followed by the removal of the photoresist to obtain the electric contacts (Figures 3h and 3i). A silicon oxide passivation layer of around 700 nm thickness is deposited by plasma enhanced chemical vapour deposition (PECVD) (Figure 3j) and a third photolithographic process is then carried out (Figures 3k and 3l). A wet etching process with buffered hydrofluoric acid solution (BHF) is carried out to open the passivation layer and thus expose the area of the microelectrodes and the electric contact pads (Figure 3m). Finally, the photoresist is stripped in acetone (Figure 3n).

**Figure 4.**
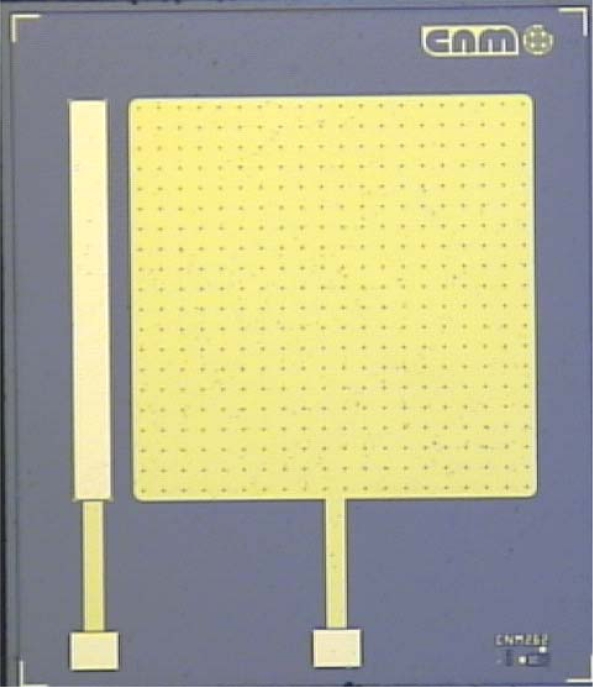
Image of an UMEA with 5 μm disk diameter and 100 μm inter-electrode distance.

Chips of 3 × 3.5 cm^2^ area containing Au or Pt disk ultramicroelectrode arrays of three different geometries have been designed and fabricated by Jiménez-Jorquera and co-workers. All of them exhibit an inter-electrode distance to diameter ratio above 10 in order to get a loosely packed array of ultramicroelectrodes under different experimental conditions as previously reported in [12]. Thus, disk units contained in an area of 2 mm × 2 mm with a diameter of 5 μm and 10 μm and an inter electrode distance of 100 μm and 200 μm, respectively, and devices having a disk diameter of 5 μm and a inter-electrode distance of 50 μm, have been designed. Chips included a counter electrode with an area of 0.7 cm^2^, separated 0.5 μm from the microelectrode. Figure 4 shows an optical microscopy image of a fabricated UMEA.

Muñoz’s group have also fabricated Au and Pt microelectrode arrays using the same standard photolithographic technology, the only difference in the fabrication process being the definition of the electrode area using an oxinitride passivation layer and a RIE process (steps corresponding to Figures 3l and 3m) [32]. Different geometries have also been studied maintaining the inter-electrode distance/diameter ratio between 10 and 20 times. Design characteristics of the UMEA fabricated by both groups at the IMB-CNM are summarized in Table 1.

In all cases, chips have been diced and fixed to a printed circuit board (PCB). Next, a wire bonding process is carried out to connect the chip electrodes to the PCB pads. UV-curable (Ebecryl 600, from Cytec Surface Specialities S.A) or thermo-curable polymers (EpoTEK H77, from Epoxy Technology) are used to encapsulate the chips depending on their final application. When using UV-curable polymer, chips with a pre-defined polymer layer deposited at wafer level are used [43]. This pre-defined layer provides a better adhesion of the polymer film used for encapsulation. In this case, a mask is used to define the window corresponding to the electrode area.

Muñoz’s group and Compton’s group, from Oxford, worked together in the characterization and development of analytical applications using UMEAs. Apart from those fabricated using photolithographic processes, whose characteristics are included in Table 1, they worked on the manufacturing of random arrays of microdisk electrodes using the patented CSIRO method. The random arrays consist of about 3,200 well-dispersed, 3.5 μm radius conducting carbon microdisks embedded in an epoxy resin [27]. They have also reported on the development of regular arrays of microdisk electrodes, arrays of microdoplets fabricated on Au substrates [32,44–48] and microband electrodes [49,50].

## 4. Characterization

Thin film UMEAs of planar configuration containing a high number of units with identical physical and chemical characteristics are commonly fabricated by standard Si/SiO_2_/metal microelectronic technology [17,29,30,51–53]. Definition of the microelectrode units is usually carried out by etching a passivation layer deposited on top of the metal layer. Provided that this step is not totally efficient, a small fraction of the electrode units within one chip remain passivated. Therefore, a thorough analytical characterization of the resulting devices and estimation of fabrication efficiency is required. For this purpose, procedures that go from simulation techniques [37], and classical electrochemical techniques, *i.e.*, cyclic voltammetry or impedance spectroscopy [54], to surface techniques such as scanning electrochemical microscopy [6,24] or microscopy image techniques [37] have been reported.

As shown in eq. (1), the electrochemical behaviour and performance of UMEAs depend on their geometry, radius and inter-electrode distance. Characterization is regularly performed by using electrochemical techniques, however with the present possibilities of computational techniques some works have been devoted to modelling the performance of UMEAs. In addition, a complete electrode characterization in terms of fabrication efficiency and analytical response combining electrochemical techniques and optical microscopy tools has been reported by our group [35].

An initial chemical/electrochemical process of the microelectrode surface is performed in order to remove residual contamination coming from chip post-processing and manipulation as described [10,33–35,37,41]. Firstly, cyclic voltammetric activation processes is carried out consecutively in a 0.1 M H_2_SO_4_ solution, in 0.1 M KNO_3_, at a potential scan rate of 0.1 V.s^−1^ in an appropriate potential window, followed by the recording of cyclic voltammetric signals in a 1 mM K_4_Fe(CN)_6_ solution, in 0.1M KNO_3_. The shape of the voltammetric curve in this solution together with the current recorded, are useful to confirm the effective removal of residual contamination and the suitable performance of the UMEA device.

Unlike a peak-shaped cyclic voltammetry response obtained with macroelectrodes, a symmetrical sigmoidal response is expected with microelectrodes. This behavior is explained due to the radial diffusion of the electroactive species towards the electrode surface as mentioned in the Theoretical considerations section. The total current can be calculated using the equation 1. Measurement of the steady state current for Au and Pt UMEAs shows a very good device-to-device reproducibility with mean current values close to the corresponding expected theoretical currents. Mean values of the yield of the fabrication process ranging from 82 to 90% for the UME units within one array chip are reported by Jimenez-Jorquera’s group. The reproducibility of the fabrication process calculated as the percentage of the relative standard deviation of the UMEA voltammetric responses is always below 6.2%. Overall, the results obtained for presented arrays fabricated by similar photolithographic processes are quite satisfactory.

**Figure 5.**
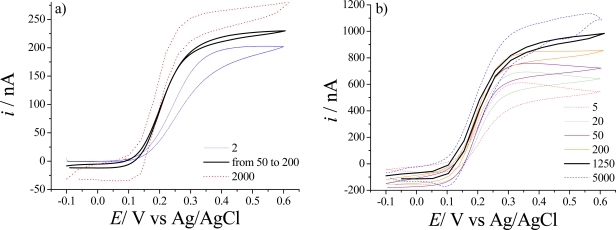
Cyclic voltammograms recorded in a solution of 1.0 mM K_4_Fe(CN)_6_.3H_2_O in 0.1M KNO_3_ at different scan rates. a) UMEA with 5 μm disk diameter and 100 μm inter-electrode distance at a scan rate of 2, 50 to 200 and 2,000 mV.s^−1^, b) UMEA 5 μm disk diameter and 50 μm inter-electrode distance at scan rate of 5, 20, 50, 200, 1,250 and 5,000 mV.s^−1^.

The influence of the scan rate on the electrochemical response is usually studied as a function of the geometric UMEA characteristics. It is shown that UMEAs with 10 and 5 μm disk diameter and 200 and 100 μm inter-electrode distance respectively devices are not greatly affected by the scan rate, this being the expected behavior when working with UMEA devices with large enough inter-electrode distances (*x* > 10) [8,12]. Nonetheless, the steady state current of UMEAs chips with 5 μm disk diameter and 50 μm inter-electrode distance varies with the scan rate, this being a clear indication of diffusion field overlap among neighboring microelectrode discs within one chip. Figure 5 clearly illustrates these differences of behavior.

**Figure 6.**
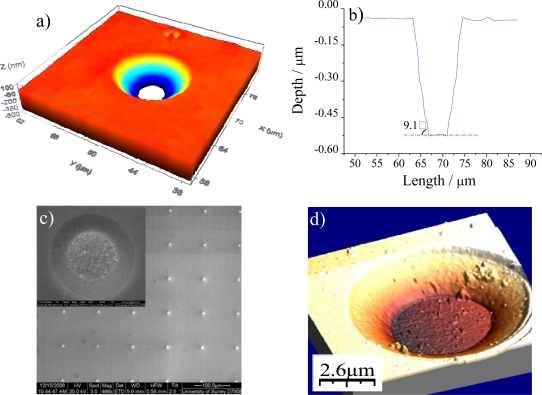
Images of a UMEA (5 μm disk diameter and 100 μm inter-electrode distance) unit obtained using a) a confocal imaging profiler; b) profile extracted from image in a). c) SEM, d) AFM.

Due to the fabrication process carried out at the IMB-CNM, UMEAs are recessed by a passivation layer of around 600 nm thickness. Here, the diffusion mode would produce a current that is smaller than that obtained with inlaid electrodes. Theoretical calculations of the recorded current could be carried out using equation 1 corrected by the factor *π* / [(4*L*/*r*) + *π*] as explained in the theoretical considerations section. However, this theoretical model did not fit the current recorded with these UMEAs. This is likely to be related to the geometry of the passivation layer side walls at the UMEA disk units, which may give rise to a diffusion regime similar to that of inlaid electrodes. The passivation layer etched by an isotropic wet process produce side walls whose cross-section shows an inverted trapezoidal shape. This structure is observed by a confocal imaging profiler as shown in Figure 6a. The side walls exhibit a small angle with respect to the substrate therefore producing behavior similar to that of inlaid electrodes.

The regular array arrangement of Pt and Au UMEAs and the inter-electrode distance (*x*) of all geometries is confirmed by SEM images. Figure 5b shows an image of a Au UMEA with 5 μm disk diameter and 100 μm inter-electrode distance as an example. SEM observation also enables the calculation of the disk diameter (*d*) for the different UMEA devices (Figure 6c, inset), all of them, being in good agreement with the theoretical values. The morphology of the UMEA surface is also analyzed by AFM images. The roughness index of the gold UMEAs units (1.0 ± 0.2 nm, n = 5) indicates a relative smooth surface. Figure 6d shows a three dimensional image of the above UMEA device, which clearly reveals a recessed electrode with a conical profile. The calculated depth values are in good agreement with those obtained by confocal microscopy showed in Figure 6b.

The 20 μm and 10 μm diameter disks of UMEA devices can be observed with the naked eye upon close examination. However, a magnifying glass or microscope is needed to see the 5 μm disks clearly and, in any case, determine the number of passivated units. A thorough analysis of the yield of the UMEAs fabrication process can be corroborated by imaging techniques based on either alteration or modification of UMEA metal surface.

One approach for the estimation of the passivated electrode units consists of the direct electrochemical oxidation of the UMEs that enables their simple observation by transmission light microscopy. However, as it is a destructive approach, a non-destructive protocol has been implemented based on the electrodeposition of gold nanoparticles (GNP) at the electrode surface. GNPs are electrodeposited onto the UMEA surface by applying a constant positive potential of +1.6 V (*vs.* Ag/AgCl pseudo-reference electrode) for 10 min in a 20 nm commercial gold nanoparticle solution. This procedure does not only enable the estimation of the number of non-passivated UMEs by transmission light microscopy, but also increase up to 100-fold the UMEA surface area without altering its electrochemical behaviour. Other protocol that enables a good estimation of the yield of the fabrication process is carried out by fluorescence imaging of a fluorophore-thiolated oligonuclotide conjugate chemisorbed to gold nanoparticles and attached to the UMEA surface. This procedure has also demonstrated the potential usefulness of the GNP-modified UMEAs in the development of biosensor devices [55].

The results of the UMEA fabrication yield obtained with the above mentioned techniques were compared and an excellent correlation was obtained in all cases. It was shown that less than 20% of the individual electrodes within one chip remains passivated [35].

An estimation of the number of inactive discs in an array was also carried out by Muñoz and co-workers [10], by depositing copper metal onto the array surface. After this plating process the active disk units could be counted by visual observation with an optical microscope and the number of inactive discs was calculated to be 34%. This lower fabrication yield compared with the one shown above may be related with the different etching processes performed to define the electrode area (wet etching *vs* RIE processes in the former and latter, respectively).

The group of Spanish researcher Miquel Esteban and his co-workers has also characterized gold UMEAs fabricated at IMB-CNM using chronoamperometric and voltammetric techniques. They studied the experimental conditions to achieve steady-state currents, the effect of different supporting electrolytes and the role of conditioning and activation processes, and demonstrated the high potential of these devices in flow injection analysis (FIA) systems [21,56].

Computational techniques were also used to deepen in the understanding of the UMEA behaviour. In this context, Muñoz’s and Compton’s groups carried out simulation studies based on the diffusion domain approximation [10]. A two-dimensional simulation method for the cyclic and linear sweep voltammetry of regular and random arrays of microdisc electrodes has also been reported [27,32,57]. These studies showed the drawbacks of working with randomly distributed microelectrode assemblies, in which diffusion field overlapping of neighbour individual microelectrodes take place, except for those approaches containing a low density of electrodes randomly dispersed in the device.

## 5. Applications in the Environmental Field

The increasing necessity of getting more and better chemical analytical information under non conventional conditions (*i.e*., *in situ* or close to place) in the environmental field has led to the development of different chemical sensor approaches. Within these, UMEAs as well as microsensors, are presented as a powerful and cost-effective alternative to conventional analytical techniques.

The sensitive detection of copper at gold UMEAs has been reported by our group [34]. GNPs were electrodeposited onto the UMEAs surface as explained in the Characterization section. Underpotential deposition-anodic stripping voltammetry of copper (II) with such modified UMEAs was performed and showed a high increase in sensitivity (25.91 ± 1.28 nC.μM^−1^) and a broader linear range of response (0–10 μM) compared with those values obtained using bare UMEAs (7.53 ± 0.57 nC.μM^−1^ and 0–2 μM, respectively). The copper content of acid extracts of contaminated soils was successfully determined with the modified UMEAs and results are in good agreement with those obtained using the ICP-AES standard method. Overall, this work showed an alternative easy-to-use novel miniaturized device for the rapid and reliable determination of copper in soil samples whose application could be readily extended to other heavy metals of environmental interest.

More recently, GNP-modified UMEAs has also been proposed by our group as a suitable transducer platform for the fabrication of biosensors [33]. In this context, the development of an amperometric peroxidase-based biosensor for the detection of phenolic compounds was described. Comparative studies carried out with microelectrodes and UMEAs, the latter being either bare or modified with GNPs, demonstrate that GNP-modified UMEAs significantly improved the analytical performance of the resulting biosensor. Horseradish peroxidase enzyme (HRP) was covalently immobilized over the electrodes by means of a dithiobis-N-succinimidyl propionate (DTSP) self-assembled monolayer. The resulting biosensors were applied to the amperometric detection of catechol, selected as a target analyte of environmental interest, at a set potential of −0.1 V *vs.* Ag/AgCl. The resulting biosensor showed a linear response to catechol in the concentration range from 0.05 mM to 0.4 mM, with a limit of detection of 0.05 mM. The use of GNP-modified UMEAs increased the sensitivity of the described biosensor approach 3-fold and 80-fold compared with the values obtained with bare UMEA and microelectrode based biosensors, respectively, thus, demonstrating the potential usefulness of the GNP-modified UMEAs in the development of biosensor devices.

On the other hand, Muñoz’s group has presented a solid-state microrespirometer consisting on a biofilm of *Pseudomonas aeruginosa* naturally developed over a Nafion modified gold UMEA. Such device is able to give qualitative information about toxicity in water streams based on the oxygen reduction current recorded at the array. When a healthy biofilm was exposed to a toxic stream, the bacteria stopped breathing, which resulted in an immediate increase of the oxygen supply measured with the UMEA [39].

Other interesting applications, also in the environmental field, have been reported by Muñoz’s and Compton’s groups. The electroanalytical detection of trace mercury (II) at gold UMEAs was first reported [37]. The array was used in a nitric acid solution using linear sweep voltammetry where a linear response for mercury additions ranging from 10 μgL^−1^ to 200 μgL^−1^ was observed. The sensitivity and detection limit was 0.11 nCμg^−1^L and 3.2 μgL^−1^ (16 nM) respectively, using a deposition time of 30 s at −0.2 V (*vs.* SCE). The same sensitivity and limit of detection was obtained in 0.1 M and 1 M chloride media. The protocol was shown to be useful for the determination of mercury on river water samples collected from the River Cherwell (Oxford).

Finally, some analytes, which can undesirably be present in certain environmental matrices, have been detected using UMEAs. The electrochemical oxidation of hydroxide ions was monitored in the range of 50 μM up to 1 mM, the sensitivity and detection limit being the best analytical data reported for hydroxide amperometric detection [41]. WO_3_-modified platinum UMEAs enabled the direct detection of bromate, chlorate and iodate, being the detection limits 0.76 μM, 2.34 μM and 133.2 μM, respectively. The electrochemical formation of such WO_3_ layer, from hydrogen peroxide-tungstanate solutions, was described [38]. Likewise, sulfite, a common waste product coming from beverage, food and pharmaceutical industries, was determined by linear sweep voltammetry at in situ plated copper-modified gold UMEAs, with a sensitivity of 0.35 nAμM^−1^ and a detection limit of 6 μM.

## 6. Conclusions

The study of the analytical performance of UMEAs and how their attractive advantages have been profited in a variety of environmental approaches in Spain have been reviewed. This overview also includes different theoretical and analytical aspects of the UMEA fabrication and characterization processes. These devices pride themselves of being robust, with low output impedance, rapid response and mass-produced at a reasonable cost, these being features common to all microfabricated electrodes. Additional advantages such as the improved mass transport towards the transducer due to radial diffusion, the greatly enhanced faradaic to capacitive current ratios; and the very small *iR* drop, are related to well ordered ultamicroelectrode arrays. Reproducibility, high sensitivity and low limits of detection are usually achieved. In addition to their small size, UMEAs are ideal to be implemented in small probes or integrated in flow systems for on-line detection, which is particular useful to carry out user-friendly rapid on-site measurements. Overall, UMEAs could be considered as a valuable, powerful, simple, rapid and suitable cost-benefit relation tool that fulfils the requests to be implemented in environmental analytical applications and could be a real alternative to commercially available conventional electrodes, expensive instrumentation and different time-consuming methods currently applied in this field.

## Figures and Tables

**Table 1.** Geometric features of ultramicroelectrode arrays of different geometries fabricated by photolithography.

***m***	***D* / μm**	***X* / μm**	***X* / *d***	**References**
36	5	100	20	[10]
72	10	100	10	[10,32]
100	10	200	20	[33–35]
128	10	100	10	[10,36,37]
210	20	100	5	[32,38]
256	10	100	10	[36,39–41]
400	5	100	20	[33–35]
1,600	5	50	10	[33–35]
2597	5	55	11	[42]

*m*, number of ultramicroelectrode units; *d*, diameter; *x*, inter-electrode distance.
